# An ICD-Associated DAMP Gene signature predicts survival and immunotherapy response of patients with lung adenocarcinoma

**DOI:** 10.1186/s12931-023-02443-0

**Published:** 2023-05-31

**Authors:** Yuxin Wu, Kexin Li, Shuang Liang, Xiaoying Lou, Yiling Li, Danfei Xu, Yue Wu, Yuan Wang, Wei Cui

**Affiliations:** State Key Laboratory of Molecular Oncology, Department of Clinical Laboratory, National Cancer Center/National Clinical Research Center for Cancer/Cancer Hospital, Chinese Academy of Medical Sciences, Peking Union Medical College, Beijing, 100021 China

**Keywords:** Lung adenocarcinoma, Immunogenic cell death, Damage-associated molecular patterns, Signature, Prognosis, Immunotherapy

## Abstract

**Background:**

While some lung adenocarcinoma (LUAD) patients benefit long-term from treatment with immune checkpoint inhibitors, the sad reality is that a considerable proportion of patients do not. The classification of the LUAD tumor microenvironment (TME) can be used to conceptually comprehend primary resistance mechanisms. In addition, the most recent research demonstrates that the release of damage-associated molecular pattern (DAMP) in TME by immunogenic cell death (ICD) may contribute to the adaptive immune response. Currently, however, there is no such comprehensive research on this topic in LUAD patients. Therefore, we set out to investigate how to reverse the poor infiltration characteristics of immune cells and boost antitumor immunity by identifying DAMP model.

**Methods:**

In this study, ICD-related DAMP genes were selected to investigate their effects on the prognosis of LUAD. To create a risk signature using the TCGA-LUAD cohort, the univariate COX regression and the least absolute shrinkage and selection operator regression were carried out, and the results were verified in a GEO dataset. Subsequently, the multivariate COX regression was applied to establish a prognostic nomogram. And the ESTIMATE and ssGSEA algorithms were utilized to analyze immune activity and the TIDE algorithm was for responsiveness to immunotherapy. Moreover, clinical tissue samples were used to verify the differential expression of 9 DAMP genes in the signature.

**Results:**

We identified two distinct DAMP molecular subtypes, and there are remarkable differences in survival probability between the two subtypes, and patients with higher levels of DAMP-related genes are “hot tumors” with increased immune activity. In addition, 9 DAMP genes were selected as prognostic signature genes, and clinical outcomes and immunotherapy response were better for participants in the low-risk group. Importantly, according to the area under the curve (AUC) value in evaluating the efficacy of immunotherapy, this signature is superior to existing predictors, such as PD-L1 and TIDE.

**Conclusions:**

Our study suggests ICD plays an important part in modeling the TME of LUAD patients. And this signature could be utilized as a reliable predictor to estimate clinical outcomes and predict immunotherapy efficacy among LUAD patients.

**Supplementary Information:**

The online version contains supplementary material available at 10.1186/s12931-023-02443-0.

## Background

Lung cancer continues to be a challenge since it is predicted to cause 1.8 million deaths globally [1]. Nearly 85% of lung cancer cases are caused by non-small cell lung cancer (NSCLC), with lung adenocarcinoma (LUAD) being the most common histological subtype [2–4]. Patients with LUAD have recently seen significant clinical benefits from molecular-targeted therapy [5]. Sadly, most patients who receive targeted treatments like EGFR tyrosine kinase inhibitors become resistant, and their prognosis remains poor [6]. Immune checkpoint inhibitors (ICIs), the most potent immunotherapeutics for LUAD, have the potential to significantly extend patients’ overall survival (OS) and progression-free survival (PFS), making them the ideal first-line treatment for advanced LUAD. However, only a small percentage of LUAD patients can benefit from ICI therapy, and the overall response ratio of ICIs continues to be underwhelming [7, 8]. PD-L1 expression, tumor-infiltrating lymphocytes (TILs), and dendritic cells (DCs) are all associated with LUAD response to ICIs [9, 10].

Immunogenic cell death (ICD), a distinctive type of regulated cell death, can elicit immunological responses specific to exogenous or endogenous antigens released by dying cells, particularly cancer cells [11]. Accumulating preclinical models and clinical trials have confirmed ICD as an essential predictor of potent antitumor immunity [10, 12], and it could be induced to sensitize LUAD patients to ICI treatment [13, 14], indicating that ICD-related biomarkers may serve as immunotherapy prognostic indicators. Integrating multiple immunological signaling pathways, such as danger signals, effector T-cell infiltration/activity, and numerous other pathways, into a unified paradigm is another remarkable feature of ICD for biomarker discovery [15–17]. The primary immunogenic feature of ICD is the production of the damage-associated molecular patterns (DAMP) [18]. Endogenous DAMP biomolecules are released, secreted, or exposed on the cell surface by dying, damaged, or stressed cells to mediate robust immunomodulatory effects [15]. Due to these DAMP, DCs might be activated and migrated, which in turn prime T cells for systemic antitumor immunity, and create long-term immunologic memory [19]. These studies highlight the critical function of ICD-associated DAMP in antigen presentation, the tumor microenvironment, and the activation of adaptive immunity. Consequently, to effectively facilitate immunotherapies for LUAD patients, it is crucial to examine DAMP gene expression patterns, and also to comprehend the link between various DAMP-related molecules and the tumor microenvironment (TME) to figure out how to improve immune infiltration and boost antitumor immunity. However, currently, there is no such comprehensive research on this topic in LUAD patients.

In this research, we developed molecular subtypes of DAMP. Moreover, we constructed and verified a novel ICD-associated DAMP risk model and prognosis evaluation in LUAD patients, and to identify immunotherapy responsiveness. In addition, the connection between this signature and the immune-related landscape of the TME in LUAD patients was thoroughly examined. Based on these findings, we may conclude that the prognosis and advantages of immunotherapy for LUAD patients could be precisely predicted with the help of this risk signature.

## Methods

### Data and clinical specimen collection

33 different cancer types’ genomic, transcriptomic, and clinical data were obtained from The Cancer Genome Atlas (TCGA, downloaded from the UCSC Xena repository). For each tumor and its control sample, the RNA-seq data were normalized as log2(FPKM + 1). The training cohort comprise data from 526 TCGA-LUAD patients, 513 of whom had follow-up information, and 353 patients had accessible clinicopathological characteristics. Affymetrix microarray information for LUAD cohorts GSE31210 (n = 246) [20] was used as an external validation set, which was collected from the Gene Expression Omnibus (GEO) database. The expressions of the corresponding proteins were examined in paired LUAD tumor and adjacent non-tumor tissues using the Clinical Proteomic Tumor Analysis Consortium (CPTAC) database. The survival information of 12 types of cancer was acquired from PRECOG tools [21]. And the flowchart was created with BioRender.com.

35 pairs of lung cancer and adjacent non-tumor tissues were collected, and all patients underwent surgical resection at the Cancer Hospital of Chinese Academy of Medical Sciences and Peking Union Medical College. All samples were collected immediately after surgery and stored at − 80 °C. The ethics committee of Peking Union Medical College Cancer Hospital in Beijing, China, approved this study in accordance with the Declaration of Helsinki of 1975. Grant number: NCC2021C-527.

### Consensus clustering analysis of DAMP

Based on DAMP-related gene expression, the “ConsensusClusterPlus” R package was used to conduct clustering analysis. The ideal cluster numbers between k = 2 and k = 9 were then evaluated and repeated 1,000 times to ensure stable results. A cluster map was also created using the R tool “pheatmap.“

### Identification of differentially expressed genes (DEGs) and biological processes and enriched signaling pathway

The DAMP-related subtypes’ DEGs were evaluated using the “Limma” R package (log2foldchange > 1, false discovery rate (FDR) < 0.05). The “clusterProfiler” R package was used to conduct functional annotation studies of the Gene Ontology (GO) and Kyoto Encyclopedia of Genes and Genomes (KEGG). The involved key pathways were identified through gene set enrichment analysis (GSEA). For pathways with FDR values of less than 0.25 and normalized P values of less than 0.05, statistical significance was found. The enriched pathways (NESs) were selected using the ranks of the normalized enrichment scores.

### Construction of the DAMP-related signature

The DAMP-related genes that were statistically significant in the univariate Cox regression analysis were then subjected to the LASSO regression analysis in order to precisely determine the coefficient values of each established association. By combining normalization and variable selection, the widely used regression technique known as LASSO improves the statistical model’s interpretability and predictive power. The DAMP-related risk model was then constructed using the LASSO–Cox regression coefficients for each gene. Using the following formula, we developed the risk signature:

$$Risk\;score = {\mathop \sum \limits^n _{i = 1}} Coefficient \; (i) \times  Expression\; of\; gene\; (i)$$

The expression of the gene (i) is the expression value of the gene (i) for each patient, and the coefficient of the gene (i) is the gene’s regression coefficient. Using the survival R package’s “predict” function, the risk value for each LUAD patient was computed, and patients were distributed into low-risk and high-risk groups by the median risk value.

### Prognostic potential and accuracy of the DAMP-related signature

The TCGA dataset was used to evaluate the prognostic value of the DAMP-related signature, and an independent GEO dataset (GSE31210) was used to verify it. The survival R package was used to examine the KM curves and estimate differences in OS, PFS, or DFS between the low-risk and high-risk groups. The consequences of the univariate Cox investigation were addressed by a backwoods plot. The Sangerbox tool was used to create a Sankey diagram [22]. Using the R package “survivalROC,“ a time-dependent receiver operating characteristic (ROC) curve analysis was also developed to ascertain the risk signature’s sensitivity and specificity. The ROC effect was calculated using the value of the area under the curve (AUC).

### RNA isolation and RT-qPCR

Total RNA was extracted from Formalin-fixed paraffin-embedded samples from the LUAD and matching tissues using TRIzol reagent (Invitrogen, Carlsbad, CA, USA). The quantitative real-time polymerase reaction assay was carried out using Taq Pro Universal SYBR qPCR Master Mix (Vazyme Biotech Co., Ltd) on a Roche LightCycler 480 real-time PCR system (Roche Diagnostics). The PrimeScript 1st Strand cDNA Synthesis Kit (Takara Bio, Shiga, Japan) was then used to reverse transcribe complementary DNA. The target gene mRNA levels were normalized to beta-actin mRNA levels for each well using the 2 ^− ΔΔCt^ method.

### Construction and validation of the nomogram model

A prediction nomogram was produced using the “RMS” program utilizing the clinical parameters and risk score. The sum of the values of each variable in each sample and each component in the nomogram formula was used to get the overall value. Calibration curves were employed to ascertain the nomogram prediction and clinical observation consistency at one, three, and five years for OS, PFS, or DFS. Furthermore, ROC curves for one-, three-, and five-year survival were used to assess the nomograms. The nomogram’s predictive power was also assessed using the concordance index (C-index).

### Assessment of immune cell infiltration

To examine the relationship between infiltrating immune cells and risk signature, we determined the immune infiltration status of the TCGA database samples using methods that are well-acknowledged. Using the R package “estimate,“ the StromalScore, ImmuneScore, and EstimateScore of each LUAD sample were calculated in terms of the stromal and immune cells’ respective gene expression patterns [23]. The single-sample GSEA (ssGSEA) method was used to quantify 28 different kinds of invading immune cells in light of the transcriptome data [24] and associated gene sets. The levels of infiltrating immune cells in LUAD between the low-risk and high-risk groups were compared using the Wilcoxon rank-sum test.

### Analysis of immunotherapy efficacy

To predict the response to ICIs, the tumor immune dysfunction, and exclusion (TIDE) algorithm was applied. When comparing the effectiveness of anti-PD1 and anti-CTLA4 treatment, the TIDE score was found to be superior to the well-known immunotherapy biomarkers (PD-L1 level, tumor mutation burden (TMB), and interferon) [25]. On the basis of normalized transcriptome statistics from the TCGA-LUAD dataset, the TIDE score was also obtained from the TIDE portal. The TIDE site was also used to gather information on the survival rate, AUC value, and immunotherapy response.

### Statistical analysis

For data analysis, R software (version 4.2.0) and the necessary packages were employed. The “survival” R program was used for survival analysis using the Kaplan-Meier curve. The two-sided log-rank test was used to analyze the differences in OS, PFS, and DFS among the major risk categories and subtypes. On these DAMP-related genes, the HR, 95% CI, and P-values were computed using LASSO-Cox regression analysis to produce the DAMP-related risk signature. The univariate Cox regression model was used to determine the DAMP-related genes that most strongly correlated with OS. To display risk prediction based on univariate Cox regression analysis, a nomogram was produced using the RMS package (version 5.1-4) of the R programming language. Multivariable Cox regression was used to investigate whether the DAMP-related risk signature and other clinical characteristics were independent prognostic factors. The calibration plot, a common chart used to evaluate a nomogram’s consistency, was also created using the RMS package. The findings were deemed statistically significant when the P-values were less than 0.05.

## Results

### The landscape of ICD-related DAMP molecules in pan-cancer

**Fig. 1:**
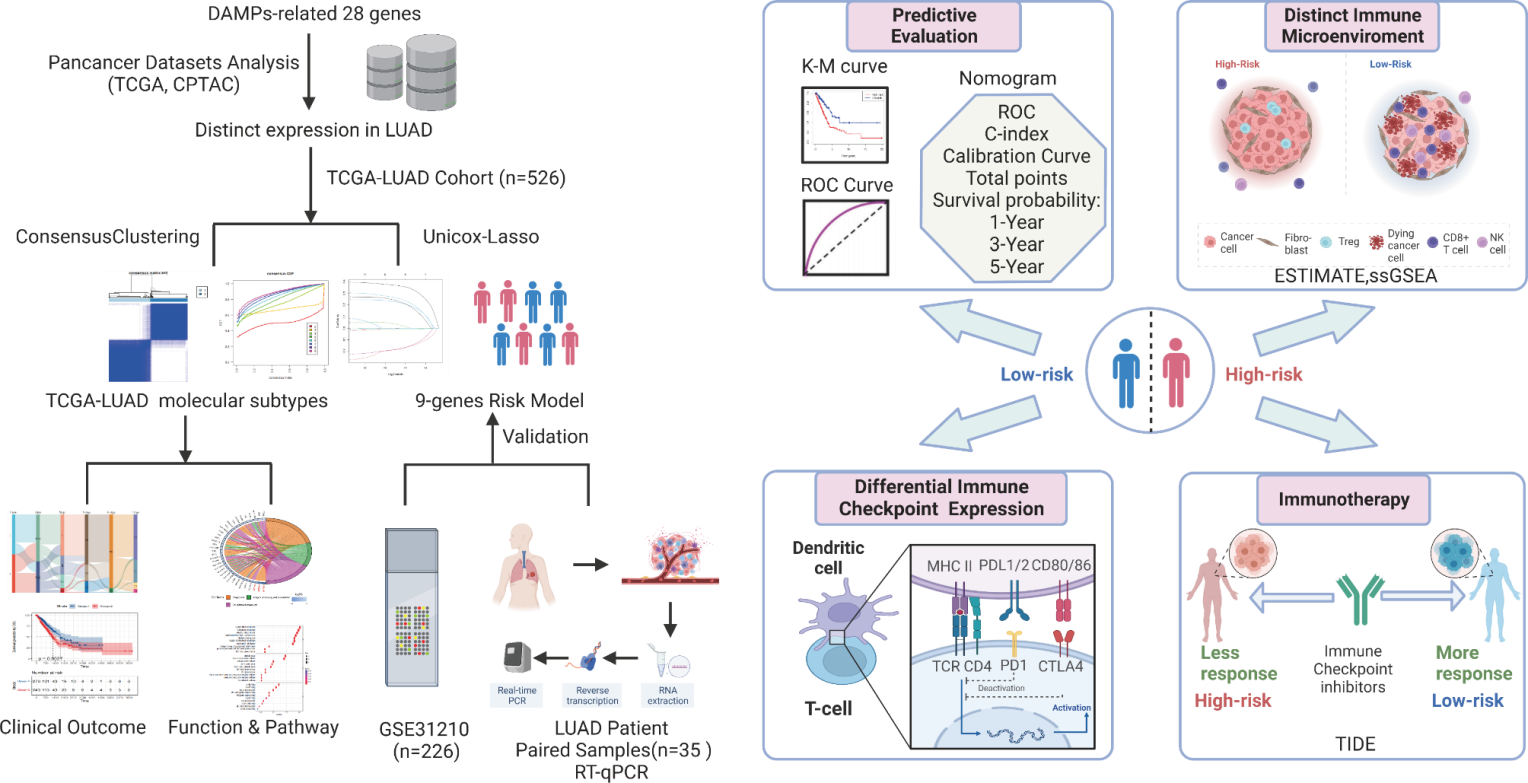
The Workflow of DAMP-related subtypes and the risk signature for patients with LUAD

**Fig. 2:**
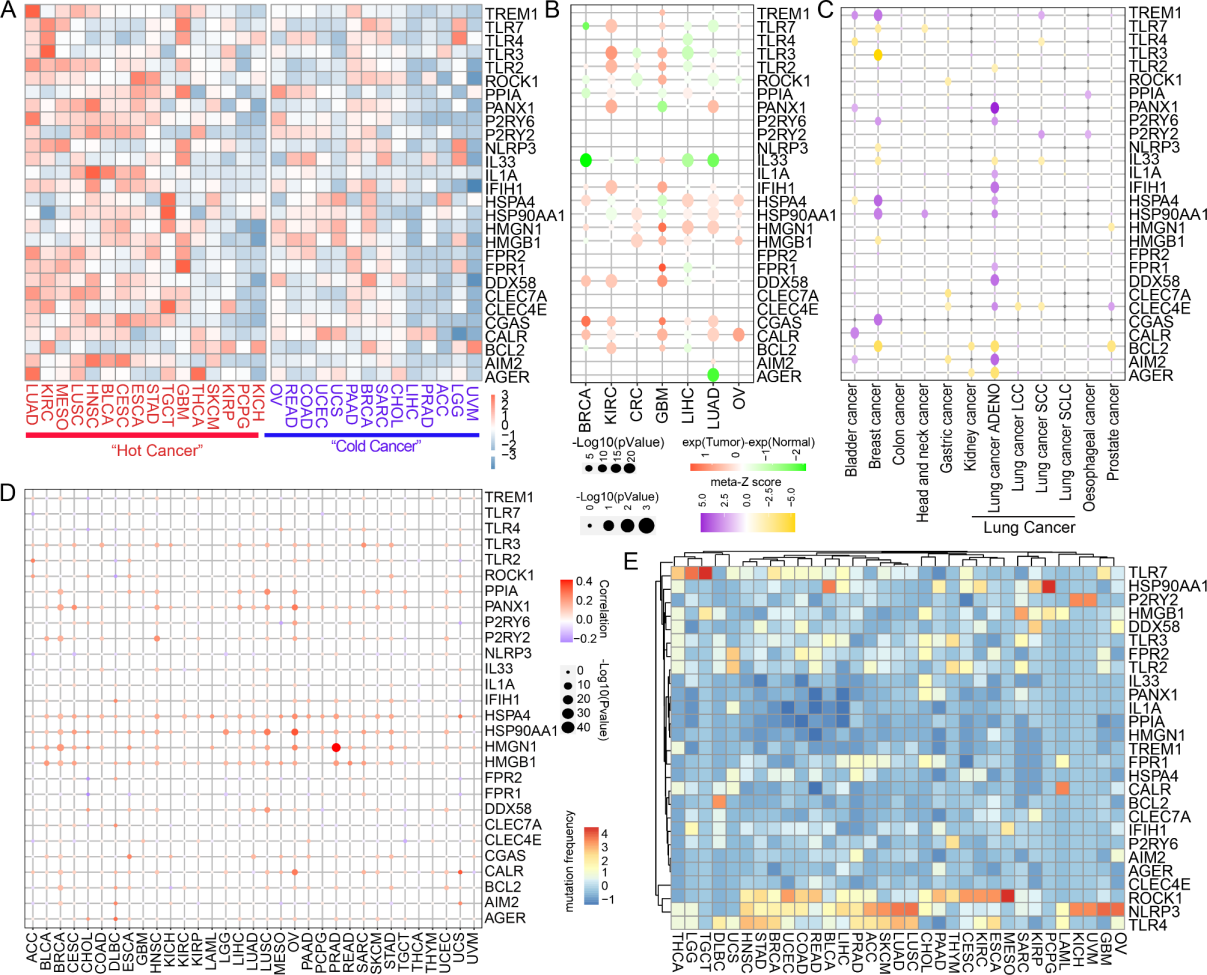
Pan-cancer analysis of 28 DAMPs-related genes. (**A**) Heatmap of RNA expression alterations of the 28 DAMPs-related genes in 33 cancer types. RNA expression levels were calculated as log2 (average expression of the tumor). (**B**) The bubble plots showing the different protein level of 28 DAMPs-related genes in 7 types of cancer in CPTAC datasets. The color of the dots represents the protein level of genes that are calculated as the average protein expression of tumor to normal. Redder dots represent higher expression in cancer tissue. Greener dots represent higher expression in normal tissue. The size of the bubbles indicates the –log10 (p-Value). (**C**) The color of the dots represents the prognostic value of 28 genes in pan-cancer. Purple dots represent favorable genes. Yellow dots represent poor genes. The size of the bubbles indicates the – log10 (p-Value). (**D**) The bubble chart shows the correlation between CNV and mRNA expression level. Red indicates positive correlation, blue indicates negative correlation. The deeper color indicates spearman correlation coefficient. The bubble size indicates the – log10(p-Value). (**E**) Mutation frequency of the 28 DAMPs -related genes across 33 cancer types. P values were calculated using Spearman correlation test or Wilcoxon rank sum test (*P < 0.05; **P < 0.01; ***P < 0.001)

The graphic workflow (Fig. 1) depicts the main design of the present research. A total of 28 DAMP-related molecules of ICD were summarized from previous studies [11, 18, 26]: AGER, AIM2, BCL2, CALR, CGAS, CLEC4E, CLEC7A, DDX58, FPR1, FPR2, HMGB1, HMGN1, HSP90AA1, HSPA4, IFIH1, IL1A, IL33, NLRP3, PANX1, PPIA, ROCK1, TLR2, TLR3, TLR4, TLR7, TREM1, P2RY6, and P2RY2. We acquired the RNA expression levels of DAMP in 33 cancer types from TCGA (Fig. 2A). According to a previous report [27], cancers were also categorized as “hot cancer” and “cold cancer” to distinguish between cancers with high- and low-immune activity. Interestingly, ICD-related genes showed higher mRNA expression in “hot cancers” than in “cold cancers”. Then, we analyzed the differential protein and mRNA expression alterations of 28 DAMP-related genes in pan-cancer (Fig. 2B and Additional file 1 Fig. 1A).

Furthermore, we investigated the prognostic potential of ICD-related DAMP molecules in various types of cancer using PRECOG tools. The findings demonstrated that genes associated with ICD may have a notable impact on the clinical outcomes of lung adenocarcinoma (Fig. 2C). The expression of ICD-related DAMP molecules and genetic variation were then found to be correlated. In most types of cancer, there was a positive correlation between copy number variation (CNV) and mRNA expression levels (Fig. 2D). We also looked into the DAMP somatic mutation frequency (Fig. 2E). According to our findings, the expressions of ICD-related DAMP biomolecules varied greatly across cancer types.

### Novel subtypes of LUAD patients identified by unsupervised learning

**Fig. 3:**
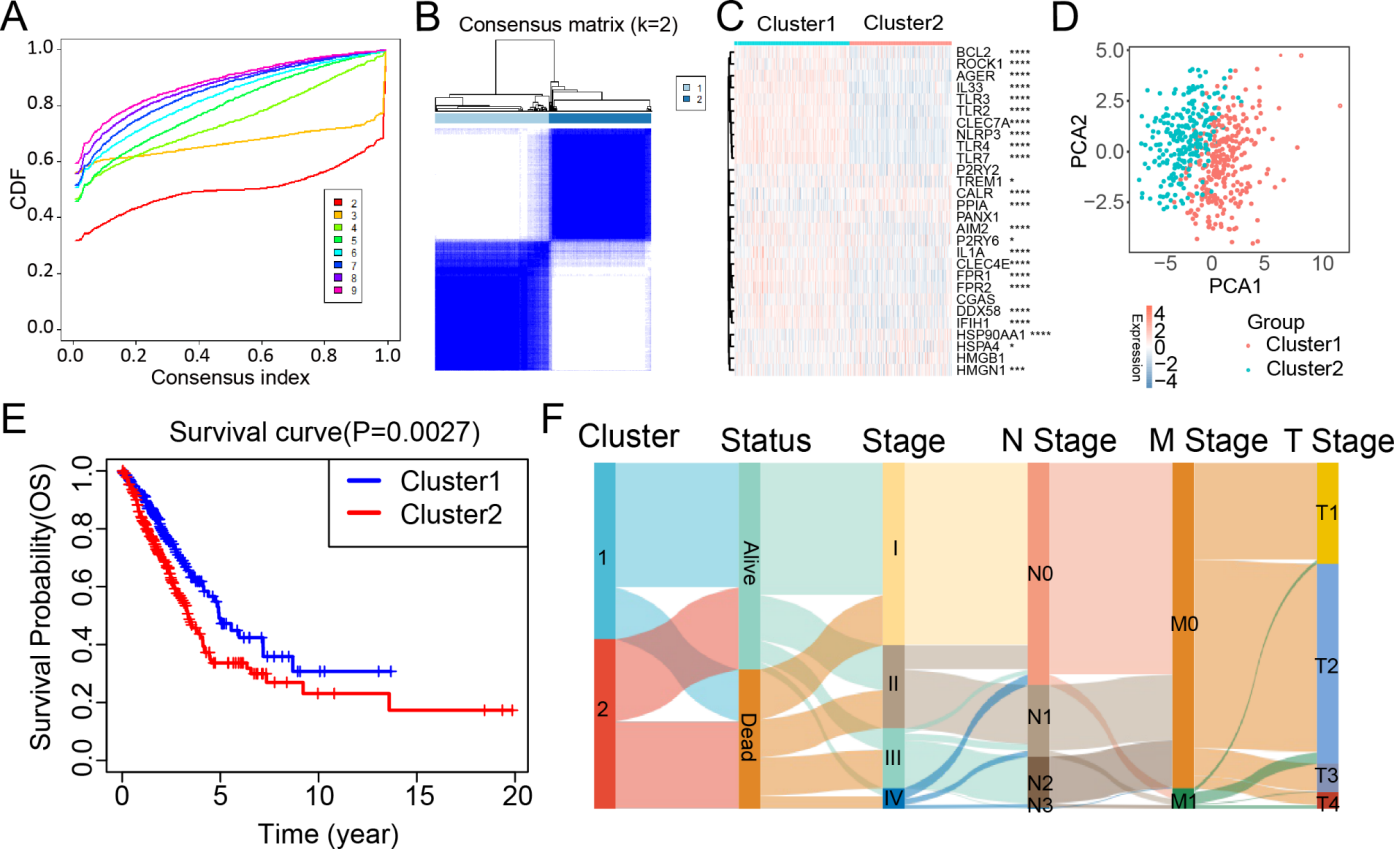
Consensus clustering of DAMP-related genes in LUAD. (**A**-**B**) K = 2 identified as the optimal value for consensus clustering. (**C**) PCA analysis displaying the gene expression distribution in TCGA-LUAD cohort. (**D**) Heatmap demonstrating gene expression profiles of 28 DAMP genes in two subtypes. (**E**) Kaplan-Meier curves of OS between the two subtypes in the TCGA-LUAD cohort. (**F**) A Sankey plot showing the distribution of clinical stage and subtypes of DAMP. P values were calculated using Wilcoxon rank sum test. (*P < 0.05; **P < 0.01; ***P < 0.001)

Intending to explore the predictive potential of DAMP genes in 526 LUAD patients, we identified two DAMP-related subtypes via unsupervised clustering using the R package ‘ConsensusClusterPlus’ with optimal clustering stability at K = 2 (Fig. 3A-B).

**Fig. 4:**
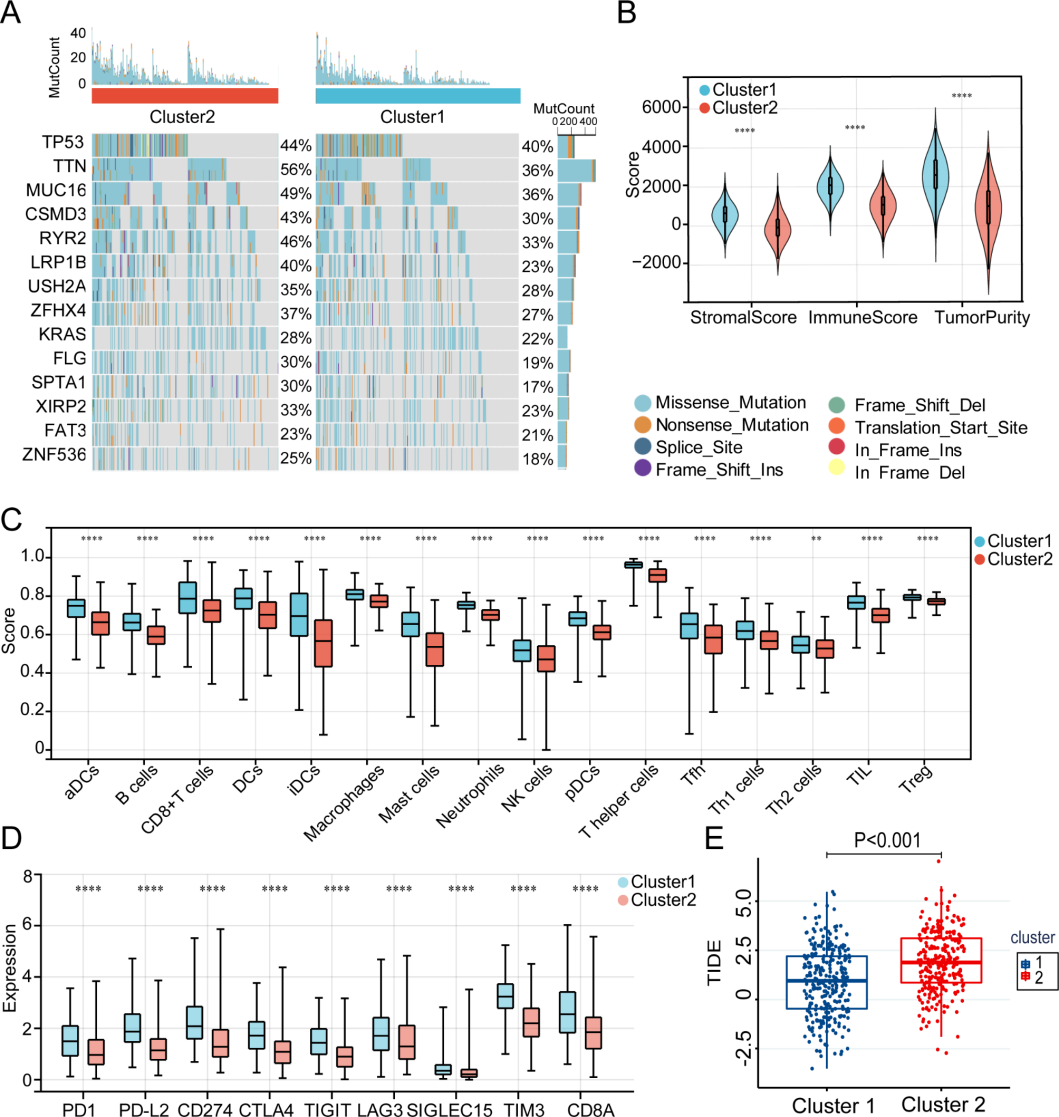
Comparison of the somatic mutation and immune landscape between DAMP-related subtypes. (**A**) Distinct somatic mutation profiles among the two subgroups. ns, not significant. (**B**) Estimation of immune scores, stromal scores, and tumor purity between two subgroups using the ESTIMATE algorithm. (**C**) Evaluation of immune cell proportions in two subgroups using the ssGSEA algorithm. (**D**) A Boxplot demonstrating gene distinct expression profiles of immune checkpoint genes in two subgroups. (**E**) Comparison of the TIDE score in two subgroups. P values were calculated using Wilcoxon rank sum test (*P < 0.05; **P < 0.01; ***P < 0.001, ****P < 0.0001)

Cluster 1 included 278 cases and Cluster 2 included 248 cases. There was a significant variance in the DAMP molecules’ expression levels between the two distinct clusters (Fig. 3C). In addition, principal component analysis confirmed that the 28 DAMP molecules’ expression levels distinguished the two clusters (Fig. 3D). Then, we conducted Kaplan–Meier (KM) survival analysis and found patients in Cluster 2 had strikingly worse OS (log-rank test; P = 0.0027) than patients in Cluster 1 (Fig. 3E). In addition, the relationship between DAMP-related subtypes and clinical factors in the TCGA cohort was visualized (Fig. 3F). Interestingly, survival status and clinical stages revealed remarkable disparities between the two subtypes, and the patients with worse survival and advanced stages were mainly included in Cluster 2. These findings indicate that DAMP-related genes could distinguish patients with LUAD based on their distinct clinical characteristics and gene expression patterns. We observed specific somatic mutation profiles between two DAMP subtypes (Fig. 4A). Cluster 2 had a higher frequency of TP53, TTN, MUC16, RYR2, CSMD3, and LRP1B mutations, though they were the most frequent mutations in the two subtypes.

According to growing evidence, ICD may increase the elicitation of antitumor immune responses. The ESTIMATE algorithm was used to evaluate the TME landscape between the two subtypes in this case. Overall, immune scores and tumor purity were significantly lower in patients in Cluster 1, indicating a higher rate of immune cell infiltration (p < 0.0001) in the TME (Fig. 4B). Next, we used the ssGSEA algorithm to compare the two subtypes’ compositions of different types of immune cells. Patients in Cluster 1 had strikingly more activated DC cells, CD8 + T cells, NK cells, B cells, CD4 + helper cells, and other immune cells (Fig. 4C). We then investigated how immune checkpoint molecules were expressed, and found that Cluster1 had high expression of PD-L1, programmed death 1 (PD-1), programmed cell death 1 ligand 2 (PD-L2), cytotoxic T-lymphocyte protein 4 (CTLA4), T cell immunoreceptor with Ig and ITIM domains (TIGIT), lymphocyte activation gene 3 protein (LAG3), sialic acid-binding Ig-like lectin 15 (SIGLEC15), and T-cell immunoglobulin mucin receptor 3 (TIM-3) (Fig. 4D). In addition, Cluster 1’s TIDE score was lower (p < 0.001) (Fig. 4E) and was correlated with a lower risk of tumor immune escape, and Cluster 1 patients had a greater likelihood ratio of benefiting from ICI treatment [25]. These results suggest that DAMP molecules may be able to distinguish the tumor immune microenvironment and that Cluster 1 was linked with the immune “hot” phenotype and Cluster 2 with the immune “cold” phenotype.

### Biological function and enriched pathway analysis of the two DAMP subtypes of LUAD

**Fig. 5:**
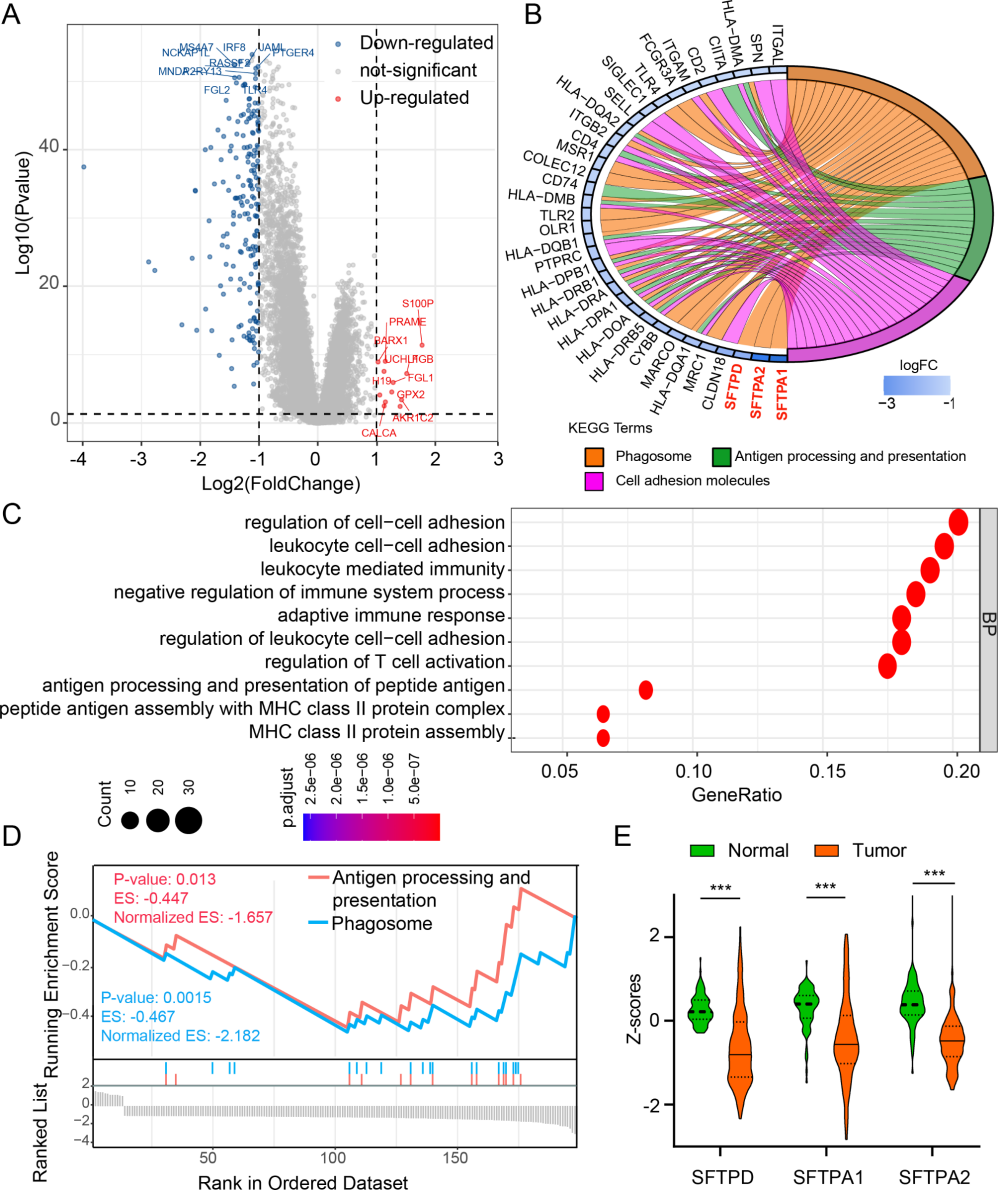
Functional analysis of DEGs based on the DAMP-related subtypes of LUAD patients. (**A**) The volcano plots displaying differentially expressed genes between Cluster1 and Cluster2 from TCGA dataset. (**B**) Chord plot depicting the relationship between genes and immune-related signaling pathways. The genes marked in red fonts refer to the most significant genes in immune-related signaling pathways. (**C**) Dots plot presents the GO signaling pathway enrichment analysis. The size of the dot represents gene count and the color of the dot represents – log10 (p. adjust-value). (**D**) GSEA enrichment plots of the Cluster1 and Cluster2 LUAD patients. (**E**) Violin plots of the protein expression of the three differential genes in normal and tumor samples in LUAD. P values were calculated using Fisher’s exact test or Wilcoxon rank sum test (*P < 0.05; **P < 0.01; ***P < 0.001)

We examined a total of 200 screened differential expression genes (DEGs) between the Cluster1 and Cluster2 subgroups in order to investigate the distinct transcriptomic signatures (Fig. 5A).

In LUAD, the GO cluster plots and KEGG plots revealed that the down-regulated DEGs in Cluster2 compared with Cluster1 were more likely to be involved in immunological signaling pathways, such as the processing and presentation of antigens, phagosomes, and cell adhesion molecules (Fig. 5B-C). Interestingly, the first three significantly differential genes, including SFTPD, STFPA1, and SFTPA2, were surfactant proteins (SP), which contribute to innate immunity and surfactant function in the lung [28]. In addition, using GSEA analysis, we showed that phagosome and antigen processing and presentation pathways were remarkably inhibited in the Cluster2 subgroup (Fig. 5D). Subsequently, we observed significantly down-regulated protein levels of SFTPD, STFPA1, and SFTPA2 in LUAD tissues (Fig. 5E). These results suggest that DAMP subtypes could potentially distinguish the tumor immune microenvironment owing to distinct innate immune activity, and could be associated with surfactant proteins in LUAD patients.

### Construction and Verification of the DAMP-related prognostic signature for LUAD patients

**Fig. 6:**
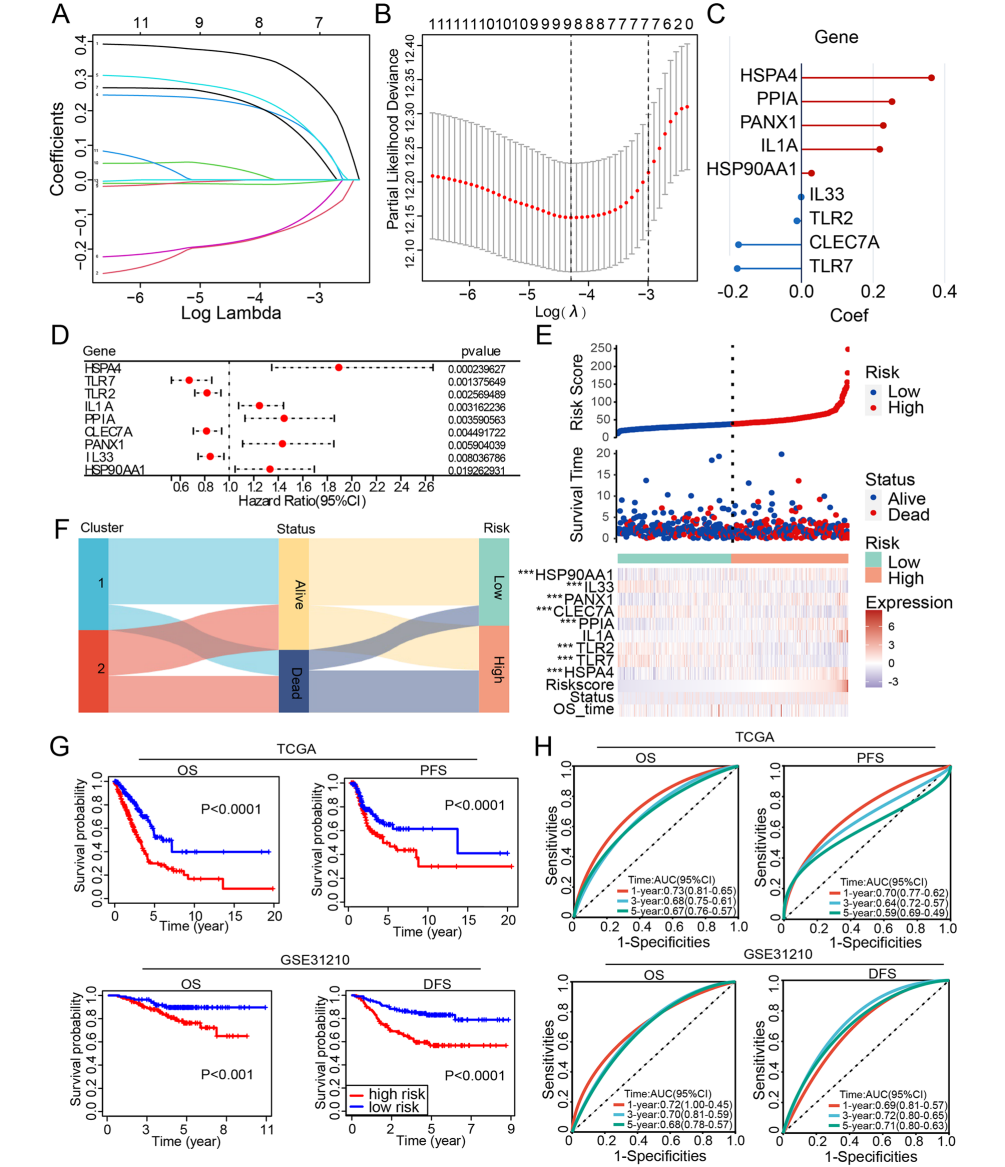
Construction and validation of the DAMP-related signature for LUAD patients. (**A**-**B**) Analysis of LASSO regression in TCGA database. The determination of “lambda” for optimal selection of gene signature. (**C**) Coefficients of the 9 prognostic molecules in the risk model. (**D**) Univariate Cox analysis evaluates the prognostic value of the DAMP genes in terms of OS; (**E**) Risk scores distribution, survival status of each patient, and heatmaps of prognostic 9- gene signature in TCGA dataset; (**F**) A Sankey plot showing the distribution of alive/dead status and subtypes of DAMP. (**G**) Kaplan–Meier analyses demonstrating the prognostic significance of the risk model in TCGA and GSE31210 cohort. OS and PFS between the two subtypes displayed in the TCGA-LUAD cohort and OS and DFS in the GSE1210. (**H**) Comparison of AUC of one-, three- and five- year OS and PFS in TCGA dataset, and OS and DFS in the GSE1210. P values were calculated using Wilcoxon rank sum test or log-rank test. (*P < 0.05; **P < 0.01; ***P < 0.001)

To construct a gene-associated prognostic model, we firstly analyzed the prognostic role of 28 genes in lung adenocarcinoma using univariate Cox regression. The results showed that the expression levels of 13 genes were associated with the prognosis of patients with lung adenocarcinoma (Additional file 1 Fig. 2). Next, the LASSO analyses of 13 DAMP-related genes from the TCGA-LUAD cohort revealed the best risk signature for estimating LUAD patients’ prognoses (Fig. 6A). The confidence interval for each lambda was also shown (Fig. 6B). Nine DAMP-associated genes, including CLEC7A, PPIA, PANX1, TLR7, TLR2, HSP90AA1, HSPA4, and IL33, were selected from the algorithm with an ideal λ value. The regression coefficients of the formula for 9 genes were displayed (Fig. 6C). The hazard ratio (HR) for four genes (TLR7, TLR2, CLEC7A, and IL33) was less than one, whereas the HR for five genes was greater than one (HSPA4, IL1A, PPIA, PANX1, and HSP90AA1) (Fig. 6D). We investigated the expression of the corresponding proteins in LUAD tumors and non-tumor tissues to ascertain the clinical significance of the risk model. Similar to the mRNA levels, HSPA4, PANX1, and HSP90AA1 were significantly elevated in LUAD tumor tissues compared to normal tissues. On the other hand, compared to non-tumor tissues, LUAD tissues expressed significantly less protein. However, the protein expression data for IL1A and CLEC7A are not available in the CPTAC database, and PPIA showed no difference (Additional file 1 Fig. 1B). Our findings demonstrate that the expression levels of the corresponding proteins are comparable to those of the model genes.

The LUAD patients were divided into low-risk and high-risk groups using the formula and the median risk value. The OS and nine gene expression profiles of the TCGA-LUAD cohort were analyzed in order to instinctively investigate the value of the risk model’s predictive ability (Fig. 6E). The composite plot demonstrates that the protective factors IL33, TLR2, CLEC7A, and TLR7 were significantly expressed at higher levels in the low-risk subtype (p < 0.001). HSPA4, PPIA, PANX1, and HSP90AA1 expression levels were also significantly lower in low-risk patients (p < 0.001). Low-risk patients had a greater chance of survival than high-risk patients, as indicated by the survival probability. The Sankey diagram shows the correlation between DAMP-related signature and the survival status of LUAD patients (Fig. 6F), and the majority of deceased patients were included in the high-risk group and Cluster2. Then, we conducted KM survival analysis and found that high-risk patients had strikingly shorter OS (log-rank test; P < 0.0001) and PFS (log-rank test; P < 0.0001) than those with low-risk patients. Additionally, these results were verified with an external cohort (GSE31210). High-risk patients had significantly worse OS (log-rank test; P < 0.001) and DFS (log-rank test; P < 0.0001) than low-risk patients (Fig. 6G), proving that the risk signature derived from these nine DAMP-associated genes was able to independently predict the clinical outcomes of the LUAD patients.

In addition, a receiver operating characteristic curve (ROC) curve analysis was carried out in order to better ascertain whether the DAMP signature is capable of accurately predicting the outcomes of LUAD patients. Results showed that the risk signature based on nine DAMP-related genes was a good predictor of survival rate. The area under the curve (AUC) value of 1-year OS and PFS reached 0.73 (95%CI, 0.65–0.81) and 0.70 (95%CI, 0.62–0.77), respectively. In line with these results, the validation set GSE31210 also showed AUC values of one-year OS and DFS reaching 0.72 (95%CI, 0.45-1.00) and 0.69 (95%CI, 0.57–0.81), respectively (Fig. 6H). Additionally, three- and five-year survival rates were also calculated for the TCGA and GEO datasets, displaying the robust nature of the predictive accuracy.

**Fig. 7:**
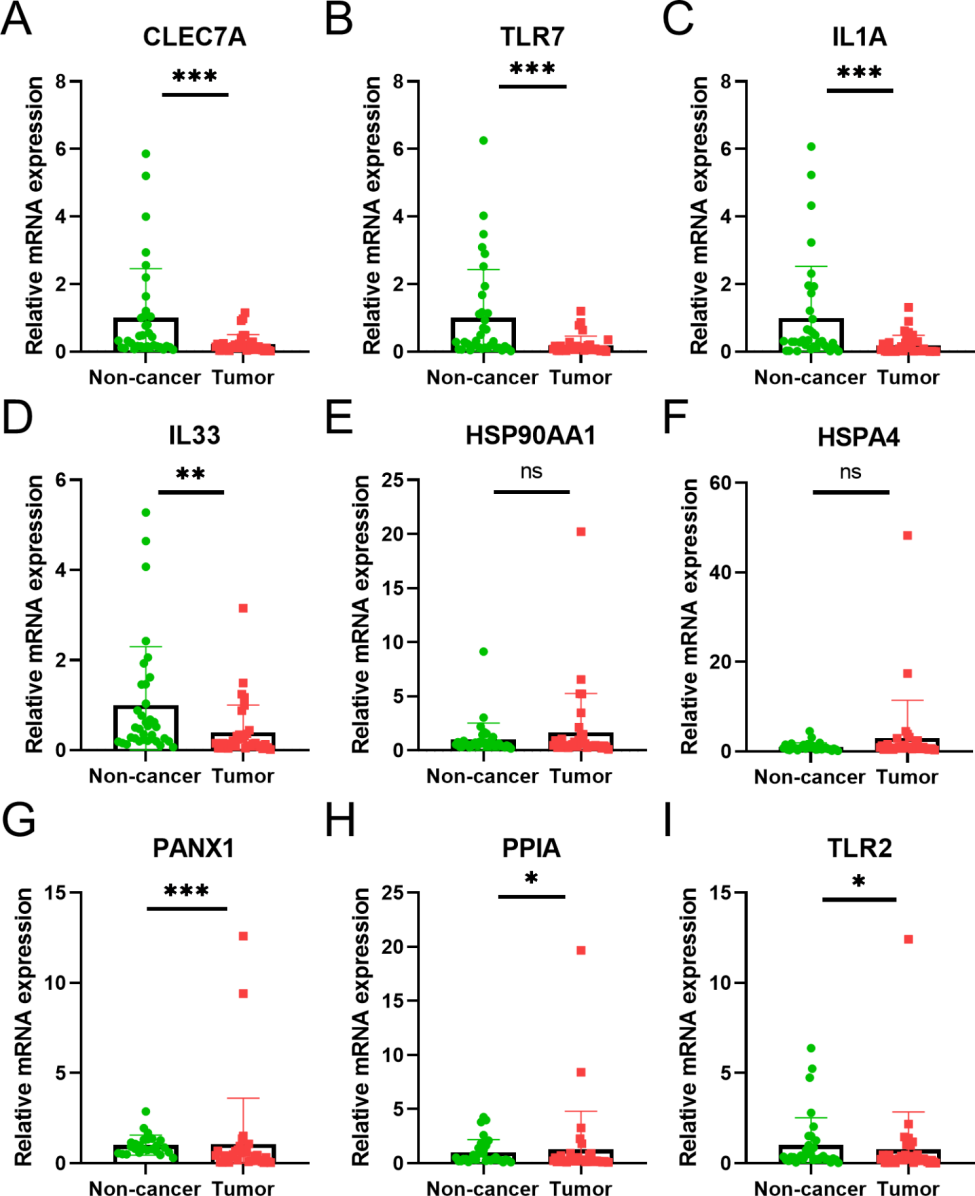
Validation of the 9 DAMP-related genes for LUAD tissues and adjacent noncancerous tissue. P values were calculated using Wilcoxon rank sum test (*P < 0.05; **P < 0.01; ***P < 0.001, ns, not significant)

To confirm the important role of 9 genes in the prognosis signature, we explored the differential expression of 9 DAMP-associated genes between 35 pairs of lung cancer tissues and adjacent noncancerous tissues. Similar with the bioinformatics results in TCGA database (Additional file 1 Fig. 1A), our experiments confirmed the expression of CLEC7A, TLR7, IL-1A and IL33 were decreased in lung cancer. And PANX1, PPIA and TLR2 were highly expressed in tumor group (Fig. 7).

### Establishment and validation of the DAMP-related prognostic nomogram for LUAD patients

**Fig. 8:**
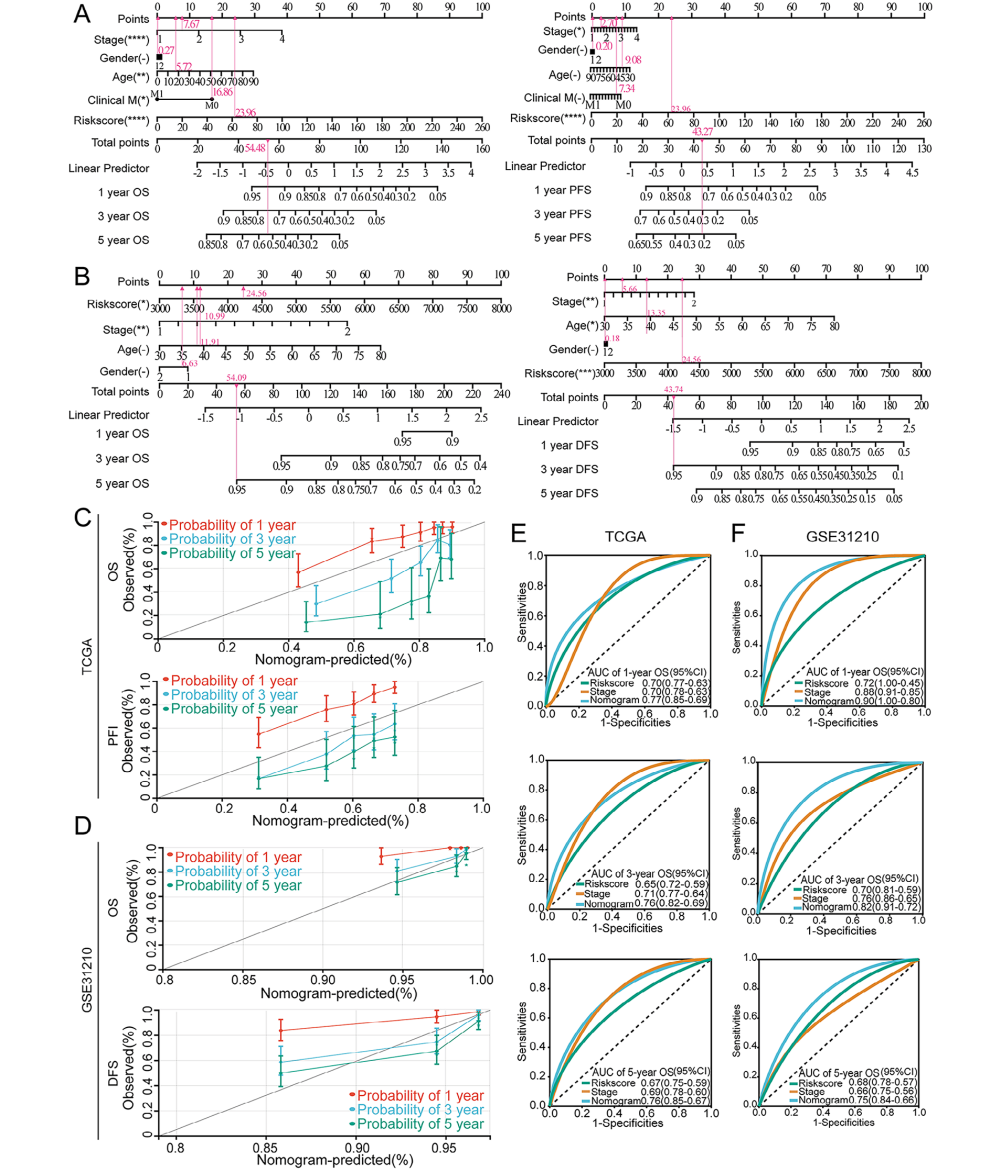
Construction of a nomogram combined with clinical characteristics in TCGA and GEO datasets. (**A**) The predictive nomogram built in combination with the risk signature and clinical characteristics predicted one-, three- and five-year OS and PFS of patients with LUAD in TCGA dataset. (**B**) The predictive nomogram built in combination with the risk signature and clinical characteristics predicted one-, three- and five-year OS and DFS of patients with LUAD in GSE31210. (**C**) The probabilities of OS and PFS at one-, three- and five- years were assessed by calibration plots of the nomogram in TCGA dataset. (**D**) The probabilities of OS and DFS at one-, three- and five- years were assessed by calibration plots of the nomogram in GSE31210. (**E**) ROC curves of the nomograms compared with clinical stage and risk score with regard to one-, three- and five-year survival in TCGA dataset. (**F**) ROC curves of the nomograms compared with clinical stage and risk score with regard to one-, three- and five-year survival in GSE31210. OS, overall survival; PFS, progression-free survival; DFS, disease-free survival

By combining common clinical factors with the gene signature, we produced a prognostic nomogram. Our ultimate objective was to develop a quantitative algorithm that could estimate the survival probabilities of LUAD patients. To estimate one-, three-, and five-year OS and PFS, individual scores based on each factor (gender, age, clinical stage, and risk score) and total scores were calculated (Fig. 8A-B).

The calibration plots demonstrated excellent agreement between the predicted probability of one-, three-, and five-year OS and the actual OS in the TCGA-LUAD cohort (Fig. 8C), with comparable outcomes for one-, three-, and five-year PFS prediction, demonstrating consistency between the actual measured values and those projected by the nomogram. In addition, GSE31210 confirmed these outcomes (Fig. 8D). Additionally, ROC analysis was used to assess the nomogram’s predictive accuracy. The AUC values of the OS nomogram were found to be higher than the risk score or clinical stage alone in both the training and validation sets, reaching 0.90 for one-year OS prediction (Fig. 8E-F), indicating that the nomograms performed better than other predictors when estimating the clinical outcomes of LUAD patients. It is also important to note that the nomogram’s stable predictive ability was demonstrated by the C-index of the TCGA dataset (OS: C-index = 0.72; PFS: C-index = 0.65), and the GSE31210 dataset (OS: C-index = 0.76; DFS: C-index = 0.73).

Based on these findings, it can be concluded that the nomogram could accurately and reliably predict the survival rates of LUAD patients.

### Prediction of immunotherapy response and tumor microenvironment landscape

**Fig. 9:**
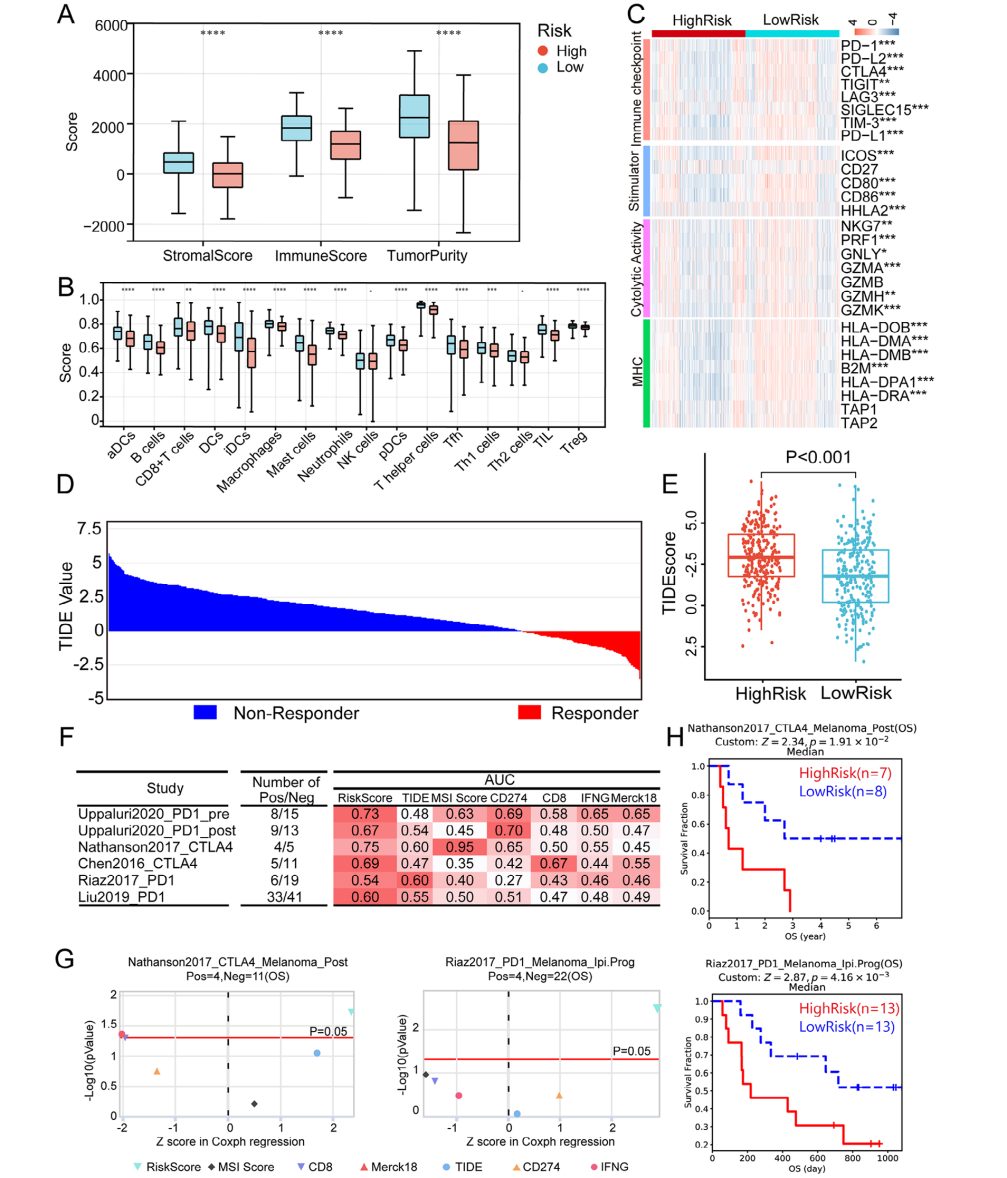
The immune landscape and the immunotherapy response of the DAMP-related signature. (**A**) Estimation of immune scores, stromal scores, tumor purity between low- and high-risk patients using the ESTIMATE algorithm. (**B**) Estimation of immune cell proportions in low- and high-risk patients using the ssGSEA algorithm. (**C**) Heat map demonstrating gene expression profiles of immune checkpoint genes, immuno-stimulator signature, cytolytic activity signature, and MHC family genes in low- and high-risk patients. (**D**) The TIDE score identifying immunotherapy response of patients in TCGA-LUAD cohort. (**E**) The TIDE score in low- and high-risk LUAD patients. (**F**) Comparison of AUC of immunotherapy biomarkers in several reported immunotherapy cohorts. (**G**) The ability of seven biomarkers to distinguish response or non-response patients with immunotherapy. Red line refers to p < 0.05. (**H**) OS probability in low- and high-risk patients of reported immunotherapy cohorts. P values were calculated using Wilcoxon rank sum test (*P < 0.05; **P < 0.01; ***P < 0.001, ****P < 0.0001)

According to the ESTIMATE algorithm analysis, patients in the low-risk group had higher immune scores, indicating a significantly higher infiltration of immune cells into the TME (p < 0.0001) (Fig. 9A). Using the ssGSEA algorithm to evaluate tumor-infiltrating immune cells, we also looked at the specific difference in infiltrating immune cells between the two risk groups. The low-risk subgroup had significantly more activated DC cells, CD8 + T cells, CD4 + helper cells, B cells, and other immune cells (Fig. 9B), demonstrating the strong link between immune infiltration and the DAMP signature found in this study. The differences in the expression of important molecules that are related to the immune system were looked at in order to determine whether or not the DAMP signature could play a significant role in the responsiveness of immunotherapy. The low-risk group had significantly higher levels of expression of immune checkpoints (PD-1, PD-L1, PD-L2, CTLA4, TIGIT, LAG3, SIGLEC15, and TIM3) and immune-stimulators (ICOS, CD80, CD86, and HHLA2) (p < 0.001) (Fig. 9C). Similar patterns were also observed in low-risk individuals for the MHC family genes (HLA-DOB, HLA-DMA, HLA-DMB, B2M, HLA-DPA1, and HLA-DRA) and cytolytic activity-related molecules (NKG7, PRF1, GNLY, GZMA, GZMH, and GZMK). The TIDE algorithm, is a stable and trustworthy ICI therapy prediction tool [25] (Fig. 9D). Interestingly, the low-risk group of LUAD patients had a lower TIDE value than the high-risk group (Fig. 9E). According to the DAMP-related signature, low-risk patients are candidates for ICI therapy because a higher TIDE score was associated with a greater likelihood of tumor immune escape and decreased benefit from anti-PD-1/CTLA4 therapy [25].

Several immunotherapy cohorts were used to test the signature’s ability to predict ICI therapy response (Fig. 9F-G). In the majority of cohorts, the AUC of the risk score for the DAMP-related signature was greater than that of the TIDE value, the microsatellite instability (MSI) score, CD274/PD-L1, CD8, and Merck18. This suggests that the risk score was better than the existing biomarkers at predicting immunotherapy response. In addition, the validation immunotherapy cohorts showed significantly higher survival rates for low-risk patients (Fig. 9H). Based on these findings, the DAMP-based signature may be able to identify patients who might benefit from ICIs.

## Discussion

Conventional cancer treatment kills tumor cells directly, whereas ICIs work by affecting tumor cells via the immune system and TME [29, 30]. The tumor immune microenvironment ought to be taken into consideration when developing predictors for ICIs in LUAD patients. Through the spatiotemporal regulation of DAMP emission, ICD can elicit adaptive immune responses that are comprehensively antigen-specific [18]. DCs, macrophages, and monocytes can drain cancer-specific antigen-filled lymph nodes thanks to DAMP’s connection to their maturation and activation [18]. An adaptive immune response to cancer is made possible by the subsequent robust response of T cells (CD8 + and CD4 + T lymphocytes) to tumor antigens. Classifying the expression of DAMP-related regulatory factors in tumor patients is essential due to the significance of DAMP in immune regulation. Our group first demonstrated that the expression of DAMP genes associated with ICD could distinguish LUAD patients who benefit from immunotherapy due to the strong connection between DAMP and the immunological microenvironment of LUAD. Additionally, we demonstrated that the expression of DAMP genes associated with ICD can distinguish LUAD patients who benefit from immunotherapy. Consensus clustering enabled us to distinguish two LUAD molecular subgroups based on DAMP gene expression. Cluster1 was associated with favorable clinical outcomes and increased levels of immune cell infiltration of B cells, CD8 + T cells, CD4 + T cells, and NK cells with strong antitumor immunity activity. Mechanistically, Cluster2 was linked to the downregulation of antigen-presenting processes, which prevented effector T cells from being activated in the antitumor adaptive immune response. There was a clear enrichment in antigen-presenting processes and signal pathways between the two subtypes as determined by the GSEA and GO analyses of DEGs. In conclusion, these findings point to the possibility of DAMP dysfunction functioning as a biomarker for the prediction of immunotherapy responses.

To further support this concept, a prognostic risk model based on nine DAMP-related genes was developed, validated, and utilized bioinformatics to classify LUAD patients into high-risk and low-risk groups. The scoring system that we developed was comprised of CLEC7A, PPIA, and PANX1. It also contained two genes from the heat shock protein family (HSP90AA1 and HSPA4), two genes from the Toll-like receptor family (TLR2 and TLR7), and two cytokines (IL1A and IL33). Despite the fact that the levels of expression and functions of these genes in the number of cancers have been determined [31–35], the clinical significance of integrating DAMP genes in LUAD patients is still unknown. In this study, our risk signature demonstrated high predictive power for survival probability and may function as an independent prognostic predictors for LUAD patients. Importantly, ICI responsiveness was accurately predicted by the risk signature derived from specific DAMP genes. The low-risk group’s favorable clinical outcomes were particularly correlated with a high level of immune cell infiltration of CD8 + T cells, CD4 + T cells, B cells, and NK cells with robust antitumor immunity. Low-risk subgroups also exhibited higher expression of immune-related genes like MHC, immune checkpoint genes, immune stimulator genes, and cytolytic activity genes. Recent research [36] indicates that patients with high levels of immune checkpoint genes may benefit from immunotherapy. NK cells are a type of lymphocyte of the innate immune system that is capable of killing cancerous cells and controlling metastatic spread [37] due to their strong cytolytic activities against tumors. MHC molecules are needed for anti-tumor CD8 + T-cell priming by DCs, according to one report [38]. The aforementioned studies have demonstrated the validity of novel DAMP-related signatures as potentially measurable prognostic biomarkers in LUAD patients, laying the groundwork for the current study’s prediction of immunotherapy. To investigate immunotherapy responsiveness, we model tumor immune infiltration by integrating the expression signatures of T-cell dysfunction and T-cell exclusion using a precise predictive model [25]. This demonstrated that low-risk patients are more likely to respond to ICIs in accordance with the DAMP signature. When comparing AUC, this signature outperformed TIDE scores, PD-L1, and CD8 in the immunotherapy cohorts that were reported.

The nomogram is frequently used to determine the prognosis of cancer [39]. Some nomograms regarding the precise prediction of LUAD patients’ prognoses have been demonstrated in previous studies [40, 41]. After combining the risk signature with other clinical characteristics, precise nomograms for OS, PFS, and DFS that are clinically applicable were developed in this study. The accuracy of the predictive nomograms in this study was confirmed by the calibration plots, AUC, and C-index.

Despite the promising outcomes, we recognize the research’s limitations. First, our findings cannot be confirmed without large-scale, multi-center clinical trials, even though this signature was examined and verified in multiple datasets. Additionally, due to the lack of prognostic information regarding immunotherapy in TCGA-LUAD patients, the immunotherapy response was evaluated using the TIDE score. To confirm the risk signature in the future, experimental studies ought to be carried out.

## Conclusions

In brief, the present study for the first time proves that the identified ICD-associated DAMP signature is a reliable biomarker for survival probability in patients with LUAD. Furthermore, the DAMP signature and the nomograms were independent prognostic predictors for LUAD. Of note, our results suggest that the DAMP signature could be an effective biomarker of responsiveness to ICIs, and demonstrated that this signature might be superior to existing biomarkers in the prediction of antitumor immunotherapy. In the future, this signature may promote distinct tumor immunophenotypes and personalized cancer immunotherapy.

## Electronic supplementary material

Below is the link to the electronic supplementary material.

**Supplementary Material 1: Supplementary Figure 1.** Pan-cancer analysis of the differential expression of the 28 DAMP-related genes. (**A**) The bubble plots showing the differently RNA level of 28 DAMP-related genes in 17 types of cancer from TCGA datasets. The color of the dots represents the RNA level of genes that calculated as average expression of tumor to normal. Redder dots represent higher expression in cancer tissue. Greener dots represent higher expression in normal tissue. The size of the bubbles indicates the ?log10(p-Value). (**B**) Violin plots of the protein expression of the 28 ICD-related DAMP genes in normal and tumor samples in LUAD. P values were calculated using Wilcoxon rank sum test (*P < 0.05). **Supplementary Figure 2.** The forest plot showing the univariate Cox regression of 28 ICD-genes in LUAD with the individual P value. The red color of P values indicates p < 0.05.

## Data Availability

The datasets supporting the conclusions of this article are available in the UCSC Xena repository (https://xenabrowser.net/datapages), GEO database (https://www.ncbi.nlm.nih.gov/geo), CPTAC database (https://proteomics.cancer.gov/programs/cptac), and the TIDE portal (http://tide.dfci.harvard.edu).

## Abbreviations

NSCLC: non-small cell lung cancer
LUAD: lung adenocarcinoma
ICIs: immune checkpoint inhibitors
TME: tumor microenvironment
ICD: immunogenic cell death
DAMP: damage-associated molecular pattern
DCs: dendritic cells
TILs: tumor-infiltrating lymphocytes
PD-L1: programmed death ligand-1
PCA: Principal component analysis
ES: enrichment scores
OS: overall survival
PFS: progression-free survival
DFS: disease-free survival
AUC: Area Under Curve
TIDE: tumor immune dysfunction and exclusion.

## References

[1] Sung H, Ferlay J, Siegel RL, Laversanne M, Soerjomataram I, Jemal A (2021). Global Cancer Statistics 2020: GLOBOCAN estimates of incidence and Mortality Worldwide for 36 cancers in 185 countries. CA Cancer J Clin.

[2] Chang JT-H, Lee Y-M, Huang RS (2015). The impact of the Cancer Genome Atlas on Lung Cancer. Transl Res J Lab Clin Med.

[3] Testa U, Castelli G, Pelosi E (2018). Lung cancers: molecular characterization, clonal heterogeneity and evolution, and Cancer Stem cells. Cancers.

[4] Little AG, Gay EG, Gaspar LE, Stewart AK (2007). National survey of non-small cell lung cancer in the United States: Epidemiology, pathology and patterns of care. Lung Cancer.

[5] Chan BA, Hughes BGM (2015). Targeted therapy for non-small cell lung cancer: current standards and the promise of the future. Transl Lung Cancer Res.

[6] Lin JJ, Shaw AT (2016). Resisting resistance: targeted Therapies in Lung Cancer. Trends Cancer.

[7] Havel JJ, Chowell D, Chan TA (2019). The evolving landscape of biomarkers for checkpoint inhibitor immunotherapy. Nat Rev Cancer.

[8] Li X, Shao C, Shi Y, Han W (2018). Lessons learned from the blockade of immune checkpoints in cancer immunotherapy. J Hematol OncolJ Hematol Oncol.

[9] Spella M, Stathopoulos GT (2021). Immune Resistance in Lung Adenocarcinoma. Cancers.

[10] Mayoux M, Roller A, Pulko V, Sammicheli S, Chen S, Sum E (2020). Dendritic cells dictate responses to PD-L1 blockade cancer immunotherapy. Sci Transl Med.

[11] Galluzzi L, Vitale I, Aaronson SA, Abrams JM, Adam D, Agostinis P (2018). Molecular mechanisms of cell death: recommendations of the nomenclature Committee on Cell Death 2018. Cell Death Differ.

[12] Wang L, Guan R, Xie L, Liao X, Xiong K, Rees TW (2021). An ER-Targeting Iridium(III) Complex that induces immunogenic cell death in Non-Small-Cell Lung Cancer. Angew Chem Int Ed.

[13] Liu P, Zhao L, Pol J, Levesque S, Petrazzuolo A, Pfirschke C (2019). Crizotinib-induced immunogenic cell death in non-small cell lung cancer. Nat Commun.

[14] Mathew M, Enzler T, Shu CA, Rizvi NA (2018). Combining chemotherapy with PD-1 blockade in NSCLC. Pharmacol Ther.

[15] Krysko DV, Garg AD, Kaczmarek A, Krysko O, Agostinis P, Vandenabeele P (2012). Immunogenic cell death and DAMP in cancer therapy. Nat Rev Cancer.

[16] Garg AD, Martin S, Golab J, Agostinis P (2014). Danger signaling during cancer cell death: origins, plasticity and regulation. Cell Death Differ.

[17] Kroemer G, Galassi C, Zitvogel L, Galluzzi L (2022). Immunogenic cell stress and death. Nat Immunol.

[18] Galluzzi L, Vitale I, Warren S, Adjemian S, Agostinis P, Martinez AB (2020). Consensus guidelines for the definition, detection and interpretation of immunogenic cell death. J Immunother Cancer.

[19] Gotwals P, Cameron S, Cipolletta D, Cremasco V, Crystal A, Hewes B (2017). Prospects for combining targeted and conventional cancer therapy with immunotherapy. Nat Rev Cancer.

[20] Okayama H, Kohno T, Ishii Y, Shimada Y, Shiraishi K, Iwakawa R (2012). Identification of genes upregulated in ALK -Positive and EGFR/KRAS/ALK -Negative lung adenocarcinomas. Cancer Res.

[21] Gentles AJ, Newman AM, Liu CL, Bratman SV, Feng W, Kim D (2015). The prognostic landscape of genes and infiltrating immune cells across human cancers. Nat Med.

[22] Shen W, Song Z, Zhong X, Huang M, Shen D, Gao P (2022). Sangerbox: a comprehensive, interaction-friendly clinical bioinformatics analysis platform. iMeta.

[23] Yoshihara K, Shahmoradgoli M, Martínez E, Vegesna R, Kim H, Torres-Garcia W (2013). Inferring tumour purity and stromal and immune cell admixture from expression data. Nat Commun.

[24] Charoentong P, Finotello F, Angelova M, Mayer C, Efremova M, Rieder D (2017). Pan-cancer immunogenomic analyses reveal genotype-immunophenotype Relationships and Predictors of response to checkpoint blockade. Cell Rep.

[25] Jiang P, Gu S, Pan D, Fu J, Sahu A, Hu X (2018). Signatures of T cell dysfunction and exclusion predict cancer immunotherapy response. Nat Med.

[26] Gong T, Liu L, Jiang W, Zhou R (2020). DAMP-sensing receptors in sterile inflammation and inflammatory diseases. Nat Rev Immunol.

[27] Lou X, Li K, Qian B, Li Y, Zhang D, Cui W (2022). Pyroptosis correlates with tumor immunity and prognosis. Commun Biol.

[28] Lin Z, Thorenoor N, Wu R, DiAngelo SL, Ye M, Thomas NJ (2018). Genetic Association of Pulmonary surfactant protein genes, SFTPA1, SFTPA2, SFTPB, SFTPC, and SFTPD with cystic fibrosis. Front Immunol.

[29] Bagchi S, Yuan R, Engleman EG (2021). Immune Checkpoint inhibitors for the treatment of Cancer: clinical impact and mechanisms of response and resistance. Annu Rev Pathol Mech Dis.

[30] Doroshow DB, Sanmamed MF, Hastings K, Politi K, Rimm DL, Chen L (2019). Immunotherapy in Non-Small Cell Lung Cancer: facts and hopes. Clin Cancer Res Off J Am Assoc Cancer Res.

[31] Beilmann-Lehtonen I, Böckelman C, Mustonen H, Koskensalo S, Hagström J, Haglund C (2020). The prognostic role of tissue TLR2 and TLR4 in colorectal cancer. Virchows Arch Int J Pathol.

[32] Michaelis KA, Norgard MA, Zhu X, Levasseur PR, Sivagnanam S, Liudahl SM (2019). The TLR7/8 agonist R848 remodels tumor and host responses to promote survival in pancreatic cancer. Nat Commun.

[33] Zhang H, Nakauchi Y, Köhnke T, Stafford M, Bottomly D, Thomas R (2020). Integrated analysis of patient samples identifies biomarkers for venetoclax efficacy and combination strategies in acute myeloid leukemia. Nat Cancer.

[34] Cohen YC, Zada M, Wang S-Y, Bornstein C, David E, Moshe A (2021). Identification of resistance pathways and therapeutic targets in relapsed multiple myeloma patients through single-cell sequencing. Nat Med.

[35] Laird DW, Penuela S (2021). Pannexin biology and emerging linkages to cancer. Trends Cancer.

[36] Yu Y, Zeng D, Ou Q, Liu S, Li A, Chen Y (2019). Association of Survival and Immune-Related biomarkers with immunotherapy in patients with Non-Small Cell Lung Cancer: a Meta-analysis and individual patient-level analysis. JAMA Netw Open.

[37] Sivori S, Pende D, Quatrini L, Pietra G, Della Chiesa M, Vacca P (2021). NK cells and ILCs in tumor immunotherapy. Mol Aspects Med.

[38] MacNabb BW, Tumuluru S, Chen X, Godfrey J, Kasal DN, Yu J (2022). Dendritic cells can prime anti-tumor CD8 + T cell responses through major histocompatibility complex cross-dressing. Immunity.

[39] Iasonos A, Schrag D, Raj GV, Panageas KS (2008). How to build and interpret a nomogram for cancer prognosis. J Clin Oncol Off J Am Soc Clin Oncol.

[40] Gong Z, Li Q, Yang J, Zhang P, Sun W, Ren Q (2022). Identification of a pyroptosis-related gene signature for Predicting the Immune Status and Prognosis in Lung Adenocarcinoma. Front Bioeng Biotechnol.

[41] Zhao F, Wang Z, Li Z, Liu S, Li S (2022). Identifying a lactic acid metabolism-related gene signature contributes to predicting prognosis, immunotherapy efficacy, and tumor microenvironment of lung adenocarcinoma. Front Immunol.

