# Supplementary material for: An ICD-Associated DAMP Gene signature predicts survival and immunotherapy response of patients with lung adenocarcinoma
Source: Respir Res. 2023 May 31;24:142. doi: 10.1186/s12931-023-02443-0 (PMC10230791; doi:10.1186/s12931-023-02443-0)
Supplement: Supplementary file 1 — Supplementary Material 1: Supplementary Figure 1. Pan-cancer analysis of the differential expression of the 28 DAMP-related genes. (A) The bubble plots showing the differently RNA level of 28 DAMP-related genes in 17 types of cancer from TCGA datasets. The color of the dots represents the RNA level of genes that calculated as average expression of tumor to normal. Redder dots represent higher expression in cancer tissue. Greener dots represent higher expression in normal tissue. The size of the bubbles indicates the ?log10(p-Value). (B) Violin plots of the protein expression of the 28 ICD-related DAMP genes in normal and tumor samples in LUAD. P values were calculated using Wilcoxon rank sum test (*P < 0.05). Supplementary Figure 2. The forest plot showing the univariate Cox regression of 28 ICD-genes in LUAD with the individual P value. The red color of P values indicates p < 0.05. [file 12931_2023_2443_MOESM1_ESM.pptx]

## Slide 1
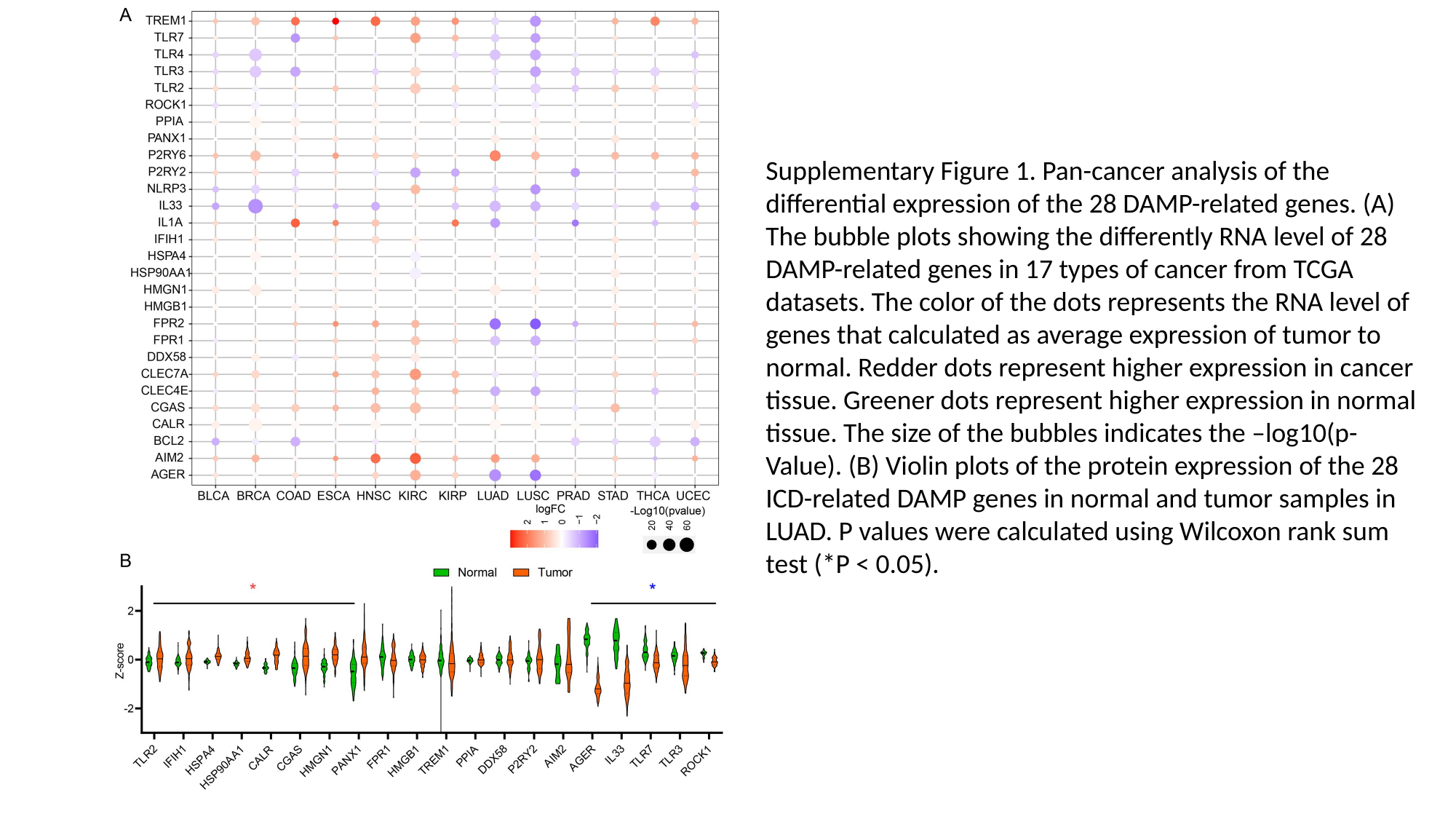

Supplementary Figure 1. Pan-cancer analysis of the differential expression of the 28 DAMP-related genes. (A) The bubble plots showing the differently RNA level of 28 DAMP-related genes in 17 types of cancer from TCGA datasets. The color of the dots represents the RNA level of genes that calculated as average expression of tumor to normal. Redder dots represent higher expression in cancer tissue. Greener dots represent higher expression in normal tissue. The size of the bubbles indicates the –log10(p-Value). (B) Violin plots of the protein expression of the 28 ICD-related DAMP genes in normal and tumor samples in LUAD. P values were calculated using Wilcoxon rank sum test (*P < 0.05).

## Slide 2
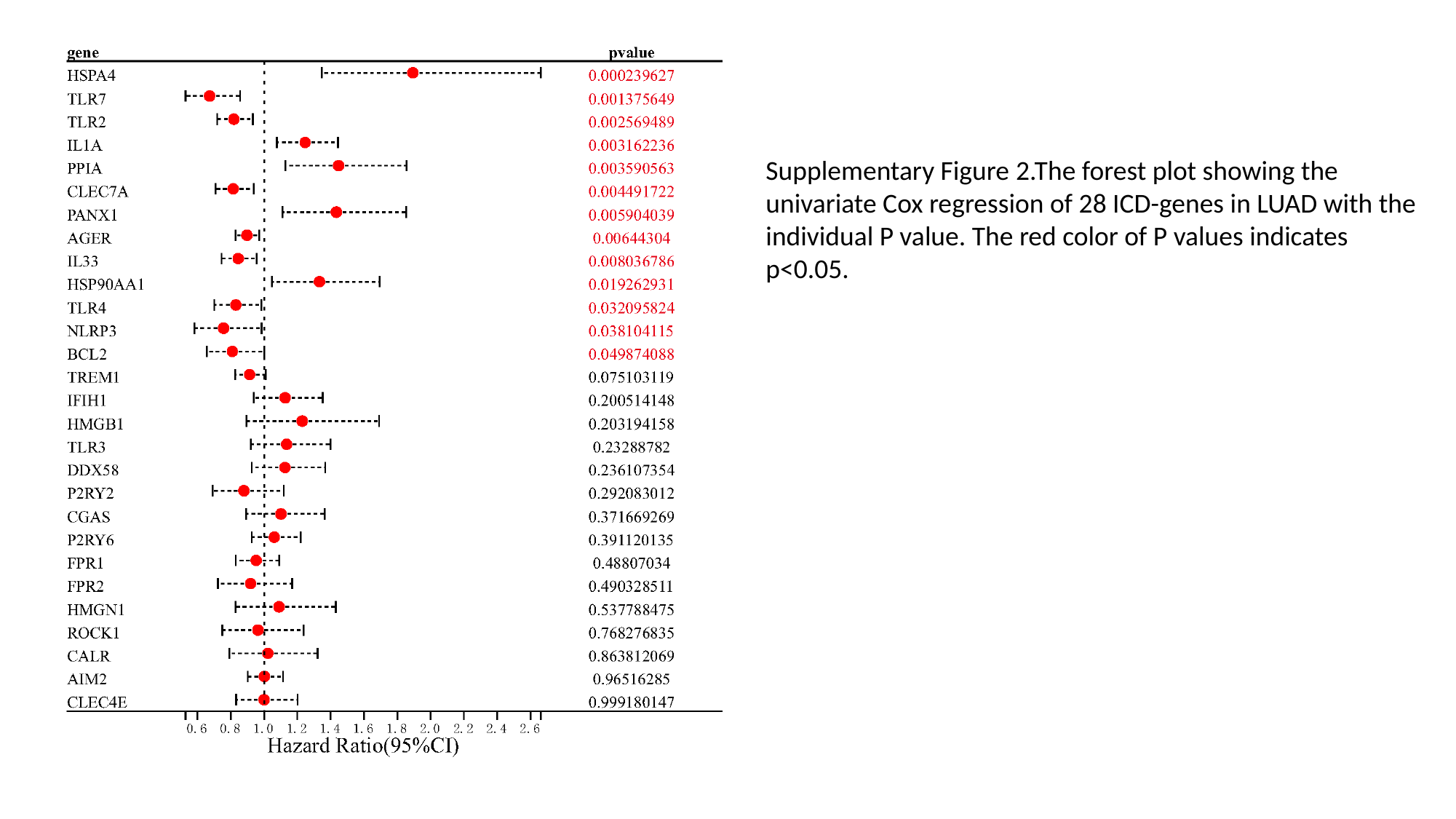

Supplementary Figure 2.The forest plot showing the univariate Cox regression of 28 ICD-genes in LUAD with the individual P value. The red color of P values indicates p<0.05.
